# Fear of Childbirth: It is Time to Talk About It!

**DOI:** 10.1055/s-0042-1758467

**Published:** 2022-11-29

**Authors:** Cibele Santini de Oliveira Imakawa, Mariane Nunes de Nadai, Monica Iassana dos Reis, Silvana Maria Quintana, Elaine Christine Dantas Moises

**Affiliations:** 1 Ribeirão Preto Medical School, Universidade de São Paulo, Ribeirão Preto, SP, Brazil; 2 Bauru School of Dentistry, Universidade de São Paulo, Bauru, SP, Brazil; 3 National Institute for Women's, Children's and Adolescent Health Fernandes Figueira, Oswaldo Cruz Foundation, Rio de Janeiro, RJ, Brazil

Fear encompasses concerns on a spectrum ranging from mild fear to phobia. 1 When it comes to fear related to childbirth, there is no consensus on its exact definition. However, there is no doubt about its importance in obstetric care. 2 3 4 When fear of childbirth is intense, it can harm the woman's health, 5 6 becoming a disabling factor that interferes with the daily routine, such as occupational and domestic activities and social life. 7

There are disagreements regarding the prevalence of fear of childbirth (FOC) due to the lack of consensus on its definition, as well as the differences in the diagnostic methods adopted by the studies. 7 8 9 In general, it is known that some degree of concern and fear can be presented by up to 80% of pregnant women. 10 11 In Brazil, this topic is rarely discussed among health professionals, and the assessment of its real prevalence remains unknown.

Some protective factors can lead to lower incidences of FOC, such as relationship stability and duration. In turn, there are risk factors that can increase its incidence, such as lack of social support; unplanned pregnancy; infertility; previous negative experiences; anxiety, depression, and other psychological disorders. 12

Younger women tend to be more afraid of childbirth than older women. The woman's nationality also seems to influence the occurrence of FOC. 13 Among the obstetric aspects, nulliparity, advanced gestational age and a history of previous cesarean section stand out as risk factors for FOC. 10 14

The preference for cesarean section is expressed by 27% of women who use the public health system and by 44% of those who access private health services in Brazil. In addition to social, economic, and cultural aspects, fear of labor pain and childbirth itself seem to be factors that could influence the mother's preference for cesarean section. 15 16 17 Besides the maternal request for cesarean section, the increase in other adverse obstetric outcomes such as preterm birth, prolonged labor, postpartum depression, and post-traumatic stress have already been related to the fear of childbirth.

It is extremely important to establish treatment plans for identified cases of FOC to minimize its negative impacts on obstetric outcomes. Accessible and timely interventions, which can contribute to the reduction of FOC, include educational and reassuring measures such as the construction of a birth plan; discussion groups with pregnant women and health professionals; access to educational materials on delivery mechanisms and obstetric care; establishment of a support network; empathic follow-up; psychological support. In more severe cases, it is essential to refer the pregnant woman to professionals specialized in mental health for better monitoring and control of symptoms.

There are still little data in the literature on the prevalence, predictive and protective factors concerning fear of childbirth according to each population, considering the obstetric history and social, economic, and cultural aspects.

A research group linked to the Ribeirão Preto Medical School of the University of São Paulo is evaluating this issue, to characterize the context of FOC in the Brazilian population and analyze the effectiveness of possible interventions to reduce the impact of this condition. Furthermore, it is intended to promote the discussion on the need for public policies aimed at identifying the fear of childbirth during prenatal care, implementing strategies to improve the education of the population regarding the physiology of childbirth and obstetric care (including information on the mode of delivery and its risks/benefits, delivery plan, labor pain management, evidence-based interventions that can occur during labor and delivery) and, consequently, expand access to these resources.

FOC exists, it is a real problem, but it remains masked by the knowledge gaps on the subject. Thus, it is essential to develop studies, establish guidelines, and train health professionals on this important issue. There is an urgent need to implement diagnostic and management measures for these cases in clinical practice. It is time to talk about it!
